# Exploring the Effect of Perceptions on Children’s Physical Activity in Varying Geographic Contexts: Using a Structural Equation Modelling Approach to Examine a Cross-Sectional Dataset

**DOI:** 10.3390/children5120159

**Published:** 2018-11-27

**Authors:** Leah G. Taylor, Andrew F. Clark, Piotr Wilk, Brenton L. Button, Jason A. Gilliland

**Affiliations:** 1 Human Environments Analysis Laboratory, Western University, London, ON N6A 3K7, Canada; ltaylo83@uwo.ca (L.G.T.); aclark2@uwo.ca (A.F.C.); piotr.wilk@schulich.uwo.ca (P.W.); bbutton3@uwo.ca (B.L.B.); 2 Department of Geography, Western University, London, ON N6A 3K7, Canada; 3 Children’s Health Research Institute, London, ON N6C 2V5, Canada; 4 Department of Epidemiology & Biostatistics, Western University, London, ON N6A 3K7, Canada; 5 Department of Paediatrics, Western University, London, ON N6A 3K7, Canada; 6 School of Health Sciences, Western University, London, ON N6A 3K7, Canada

**Keywords:** physical activity, children, Canada, urbanicity, accelerometer, barriers, perceptions

## Abstract

Most Canadian children are not meeting the recommended 60 min of moderate-to-vigorous physical activity per day. Research suggests that children’s perceptions of their environment have an influence on their physical activity behaviours, but there is a lack of generalizability among previous work. The purpose of this study was to assess the mediating effect of children’s perceptions of barriers to physical activity on the relationship between their environments and their level of moderate-to-vigorous physical activity (measured with accelerometers). Structural equation modelling stratified by gender was used to assess the research objective in a sample of 546 participants aged 8–14 years old from Northwestern and Southwestern Ontario, Canada. In both models stratified by gender, perceptions of barriers did not significantly mediate the relationship between urbanicity and physical activity. Independent of all other factors, there was no significant relationship between urbanicity and physical activity in girls, but there was in boys. These results offer insight into potential processes by which perceptions impact physical activity and provide initial information to further our understanding of the behavioural aspects of physical activity through multiple levels of analysis. Researchers must continue to improve efforts for quantifying the experience of children’s daily activity contexts.

## 1. Introduction

The ongoing trend of low levels of physical activity in Canadian children is a concern for population health [1,2], as physical activity participation is associated with many physical, mental and social health benefits [3,4,5]. Over the past two decades, research has consistently demonstrated strong evidence of positive linear relationships between type, duration, and intensity of physical activity and a variety of health outcomes, prompting recommendations for increasing regular physical activity as a health promotion and disease prevention strategy in children [3,5,6]. Physical activity, especially moderate-to-vigorous physical activity (MVPA) has been associated with benefits related to adiposity, cardiovascular health, brain development, musculoskeletal health and fitness, pro-social behaviour, academic achievement, and quality of life improvements for children and youth [2,3,5,7]. The current Canadian guidelines for physical activity recommend that children and youth (aged 5–17 years) achieve 60 min of MVPA per day to achieve these optimal health benefits [8].

Despite the wide variety of potential benefits to children’s health, only 33% of Canadian children achieve the recommended 60 min of MVPA each day [1]. Multiple factors have been associated with the achievement of physical activity guidelines, including individual-level characteristics (e.g., demographics, socio-economic status, knowledge, perceptions) and characteristics of the physical environment (e.g., urbanicity, accessibility to MVPA opportunities). While there are multiple individual-level factors that have been found to influence MVPA behaviour (e.g., ethnicity, adiposity, education/literacy), the relationship with MVPA is inconsistent throughout the literature [9,10]. In contrast, age, gender, and socioeconomic status, are individual-level factors consistently associated with children’s activity levels. Research has found that as children get older, they are less likely to be physically active and less likely to achieve physical activity standards [1,11,12]. It has been well documented that girls are less likely to be physically active than boys [1,11,12,13]. Finally, with increasing socioeconomic status, activity levels increase and sedentary time decreases [10,13,14,15].

In addition to individual-level factors, systematic reviews have identified environmental correlates with physical activity, including natural and built environments [16,17,18,19,20]. As explained by Orstad and Colleagues [21], one’s physical activity levels are also influenced by their perceptions (cognitive representation) of their physical environment. Perceptions are formed through experiential context. Context is the environment in which children live, including indicators that can be objectively measured and evaluated such as population density and built form; but context also includes the interplay of the physical, social, cultural and structural forces to which they are exposed [22,23]. Orstad et al. [21] explain that children’s perceptions of their surrounding environment are developed through a cyclical process that is interactive with social, cognitive and affective experiences. Research has indicated that one’s perceptions of their environment may be more important than the physical environment alone for predicting physical activity behaviour [21,22,23]. The purpose of this study is to assess the mediating effect of children’s perceptions of barriers to activity on the relationship between their urbanicity and MVPA.

When considering issues or opportunities that hinder/encourage physical activity, a valuable way to consider the environmental influences on children’s perceptions is through recognition of barriers to physical activity. Based on an individual’s experience of context, children living in the same physical environment may experience differences in the perception of barriers relative to their interactions with their environment. Three groups of barriers consistently demonstrate an influence on physical activity: neighbourhood, social, and safety barriers. Neighbourhood barriers usually refer to the lack of availability of and/or accessibility to physical activity resources in a child’s environment and have consistently demonstrated a negative effect on activity levels. This could include issues due to distance to facilities, transportation options, and residential density or design factors, as well as the presence/amount of age appropriate equipment/activities/landscape design features for activity in a child’s community [16,24] Social barriers are forces that exist formally and informally throughout children’s neighbourhoods and have demonstrated significant influence on the physical activity of children throughout the literature. Mechanisms of influence include parental influence, and relationships with peers positively (i.e., presence of friends) and negatively (i.e., bullying) [22,25,26,27,28,29].

Throughout the physical activity literature, safety barriers have been interpreted in various ways and have demonstrated mixed results. Barriers include presence or fear of strangers, loose animals, traffic dangers, poor neighbourhood infrastructure, and crime [24,30,31]. Beets and Foley suggest it may not be the presence of actual environmental characteristics that directly affect safety influencing physical activity levels, but rather that the perceptions of neighbourhood characteristics that promote safety may be more influential in decisions with respect to participation in physical activity [29]. Previous work by the authors [32] sought to understand how these barriers were associated with children’s environments. It was determined that 32 barriers related to safety, social relationships, and the neighbourhood were perceived to have influenced physical activity, and these perceptions differed in relation to children’s environmental contexts.

It has been well established that the environment and perceptions of barriers impact children’s MVPA [16,17,18,21], and that children perceive barriers in their environments differently [32,33]. However, there is very little known about how the perceptions of barriers to physical activity alter the relationship between the physical environment and MVPA. To fill this gap, this paper examined if children’s perceptions of barriers to MVPA mediated the relationship between children’s contexts and their MVPA behaviour. This research will provide valuable information to take a direct approach to target the MVPA of Canadian children and youth [34]. Furthermore, while research exists assessing the relationship between subjective environmental barriers to physical activity with objective physical activity, results in the literature primarily focus on populations in large urban or mid-sized cities, especially outside of Canada [16,18,24]. Based on the heterogeneous nature of the Canadian context, it is important to acknowledge the lack of generalizability of previous work to children’s health in rural areas of this country [35]. The present study aimed to address the paucity of research discussing children outside of large urban centres by incorporating a spectrum measurement tool to assess the physical environment at multiple levels of urbanicity. This is one of the first studies in physical activity literature on Canadian children to take such an approach.

On the basis of the literature and evidence reviewed, the major hypothesis of this study was that children’s perceived barriers to physical activity mediate the relationship between urbanicity of their home neighbourhood with physical activity levels. It was hypothesized that perceived social barriers would have the strongest mediating effect between the physical environment and MVPA. This is because social factors such as neighbourhood social cohesion, relationships with neighbours, and availability of spontaneous group social activities have consistently demonstrated a positive association with children’s physical activity levels in urban and rural subsamples of children [36,37]. Perceived neighbourhood and safety barriers were hypothesized to have the mediating effects to a lesser extent. While evidence of a relationship between perceiving greater barriers within these themes and experiencing lower physical activity levels does exist [22,30,38,39,40], these forces are context-specific and can change based on personal factors such as perceived self-efficacy for engaging [41], or external forces such as parental rules and local policies [25,30,31,32,33,40,42].

## 2. Materials and Methods

This cross-sectional study draws from the Spatial Temporal Environment and Activity Monitoring (STEAM) project, a multi-year mixed methods research study (2010–2016) that investigates the environmental influences on the health and well-being of children ages 8 to 14 years. The data collection took place in two study locations, in Southwestern Ontario (2010–2013) and in Northwestern Ontario (2016). Schools in Southwestern Ontario were randomly selected from groups of schools stratified by socio-economic status and urbanicity of the school environment and all schools in the Northwestern Ontario community were selected to participate. All selected schools were invited to participate and enrolled through the principal. Children in grades 4 through 8 were invited to participate in the study through classroom presentations. Children who received informed parental consent and provided their own informed assent were enrolled as participants in the study. The STEAM protocol was approved by the University Non-Medical Research Ethics Board and the respective research officers of the participating school boards. Details of the project recruitment process can be found elsewhere [30,43,44].

For each cohort of students, the data was collected over two seasons to allow for an examination of the impact of seasonality on children’s mobility and health-related behaviours. This study will focus on one season from each cohort to ensure the general seasonality is comparable between groups of children: spring (2010–2013) in the South and fall (2016) in the North. This study uses data provided by passive-global positioning system (GPS) tracking, accelerometers, and the youth survey. The GPS monitor was worn by the participants during all waking hours for up to 8 days and used in this study to identify spatially-accurate home locations for each child. Participants were also asked to wear an accelerometer, and to objectively measure their activity levels, for eight consecutive days (4–6 weekdays and 2–3 weekend days) for all waking hours, removing it only for sleeping, bathing, and swimming. Finally, participants were asked to complete a detailed survey that asked children about demographics, and perceptions about their barriers to physical activity.

The initial dataset used for this study included 1068 children from 35 schools across Ontario. Before conducting any analyses, a series of inclusion criteria were developed to ensure the quality and completeness of the observations used. The first criterion was that participants must have at least four days of accelerometer data with a minimum of 10 h of valid wear time [45], and at least one valid weekday, and one valid weekend day (*n* = 565) [46,47]. Non-wear time was classified as 60 or more minutes of motionless bouts and was excluded from the analysis [48]. The second criterion was that participants must have completed questions on the youth survey about age, gender, and perceptions of barriers to PA (*n* = 892). The final criterion was that a valid home location must be determined by the GPS data. Applying all the inclusion criteria to the dataset left a final dataset has 546 children (62% of all children in the sample) with complete data.

### 2.1. Measures

#### 2.1.1. Outcome Variable

The outcome variable used in this study was an objective measure of MVPA, defined as the average number of minutes children spend in MVPA across all valid days [8]. The outcome variable was measured using a portable Actical^®^ Z accelerometer that participants wore on their right hip (so as to not impede activity) attached with a nylon-elastic band. This device was calibrated to measure energy expenditure in 30-s epochs, providing an index of physical activity intensity throughout the course of wear time [49]. MVPA movement thresholds were defined as 1500 or more activity counts per minute [48].

While there is no consistent gold standard for minimum thresholds for measuring accurate PA, the inclusion/exclusion criteria of four valid days with at least one weekday and one weekend day are found to be an acceptable threshold in the literature [46]. A four to five day monitoring period has a test–retest reliability of 0.8 among children (grade 1 to 6), and 0.7 among adolescents (grade 7 to 12) [45]. One valid weekday and one valid weekend day is required to ensure the differences in physical activity behaviour between weekdays and weekend days are accounted for when measuring average MVPA. Requiring both types of days created a better representation of physical activity levels for each participant across an entire week [46,47].

#### 2.1.2. Exposure Variable

Previous work by Taylor et al. [32] demonstrated the need for considering more than a dichotomous urban-rural definition when analysing the influence of children’s environments on perceptions of barriers to physical activity. This study used objective measures of population density and intersection density to develop an urbanicity index, which is a spectrum approach considering the heterogeneity of built form and land uses, while providing an objective tool for classifying data [50]. The urbanicity index was created based on the sum of z-scores of both population density and intersection density around the home location for each child. Population density was measured by identifying the number of people per square kilometre within each home location’s census dissemination block. Intersection density was measured by the number of 4-way intersections per square kilometre within 500 m of each home location.

#### 2.1.3. Mediating Variables

The mediating variables in the model were children’s reported perceptions of barriers to their physical activity. These barriers were measured by the child survey, with a full list of questions found in Table 1. Survey questions were adapted from the validated Neighbourhood Environment Walkability Survey [51]. Additional questions were developed based on background relationships identified in the literature, use in previous studies, or to measure necessary socio-demographic characteristics of the participants. Four questions asked about the presence of facilitators and were reverse recoded to maintain consistency in this study (i.e., *do not* know people, *not* enough sidewalks, *not* enough bike lanes, *not* enough trees). The survey was conducted with 4-point Likert-type questions (strongly disagree, somewhat disagree, somewhat agree, strongly agree), but the Likert-type data was recoded to three thematically defined groups to assess children’s responses as in Likert scales (see Table 1). Each score has a minimum of four questions, which were combined into a single composite score for each participant to provide a quantitative interval measurement scale (i.e., 1 for *strongly disagree*, 2 for *disagree*, 3 for *agree*, 4 for *strongly agree*) [52]. This tool was used to consider the responses as continuous variables within the structural equation modelling.

#### 2.1.4. Effect Modifier and Co-Variates

The model used gender as an effect modifier. This is because the magnitude of effect of the exposure urbanicity mediated by perceptions of barriers on MVPA would vary according to a child’s gender. This is based on previous research which found girls are more likely to significantly perceive more barriers to physical activity than boys [32] and boys achieve significantly more minutes of MVPA than girls [1]. Median household income (MHHI) and age are included in the model as control variables due to their strong explanatory power with both barriers to physical activity and MVPA. MHHI (in Canadian Dollars) is measured at the Census dissemination area that a child’s home is located within. Data from the 2011 National Household survey was used for Southwestern Ontario and 2016 Census on Canada was used for Northwestern Ontario. Age, measured as a continuous variable in years, was assessed in the child survey as a demographic question.

### 2.2. Statistical Analysis

**Figure 1:**
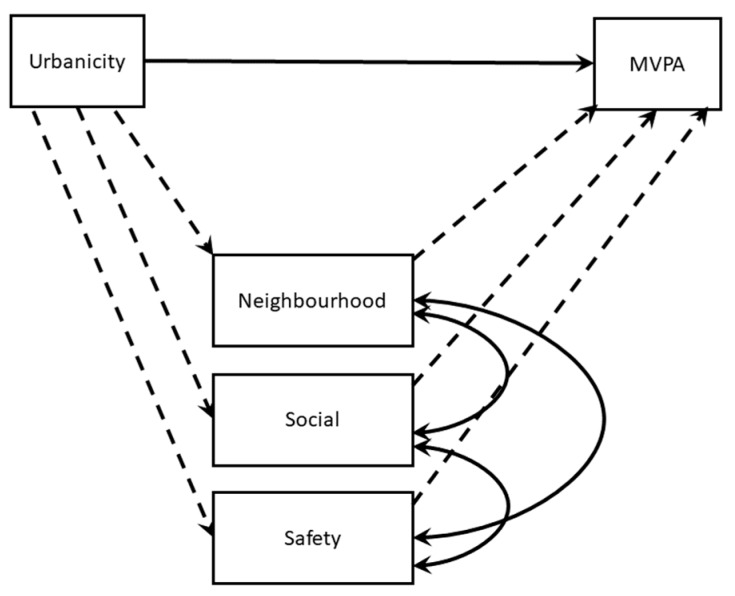
Conceptual model being evaluated with a structural equation model. In structural equation modelling (SEM), observed variables are demonstrated with squares, and relationship pathways are demonstrated with arrows. Straight arrows with a single head indicate a causal relation. A curved arrow with two heads indicates a potential association between variables. Solid lines represent direct relationships and dashed lines represent indirect relationships. MVPA: moderate-to-vigorous physical activity.

To assess the data collected from participants, structural equation modelling (SEM) was employed. SEM allows researchers to test multiple regression equations simultaneously without the assumption of a perfect relationship between all independent variables as in regression. SEM makes the assumption that all variables are additive in a linear relationship, assessing the direct and indirect effects of the variables within the model [53]. While SEM has been used in other studies examining children’s MVPA [54,55,56], this is the first study to our knowledge that has used SEM to explore the relationship between urbanicity and MVPA while accounting for perceived barriers to MVPA. Data cleaning and preliminary analyses to test the data quality were conducted in SPSS 24.0. Missing data were handled with full-information techniques. Statistical significance was determined at *p* < 0.05. Model testing was conducted in Mplus 7.4 [57]. Model fit was not tested because it was a saturated model, therefore all possible pathways were included. The SEM is testing the conceptual model in Figure 1, which was run twice with gender as the effect modifier, and controlling for age and MHHI. The dashed paths indicate indirect effects related to the primary research question.

## 3. Results

The relationships between the measured variables within the model are presented in Table 2. The specific mediating effects assessing the main research question are presented in Table 3. Results are descriptively presented separately for girls and boys below.

### 3.1. Girls

Across both Ontario study areas, there were a total of 316 participants who identified as a girl. While there are relationships between variables within the model, perceptions of neighbourhood, social and safety barriers did not significantly mediate the relationship between urbanicity and MVPA in the final model. This is demonstrated in Table 3, Model 1. Additionally, independent of all other factors, there was no significant relationship between urbanicity and MVPA in girls.

When analysed within the model, four relationships remained significant. These relationships are seen in Table 2, Model 1. MVPA was significantly negatively related to two factors. With each year increase in age, MVPA decreased by about 2.4 min (*p* = 0.04). As well, with each increase in the likelihood of reporting perception of social barriers, MVPA decreased by 4.3 min (*p* = 0.01). With increasing urbanicity, girls were significantly less likely to report perceiving neighbourhood barriers (*p* = 0.04). Finally, girls who reported neighbourhood barriers were significantly more likely to report social barriers.

### 3.2. Boys

Across both study areas, the total sample included 230 participants who identified as a boy. In the final model, as demonstrated in Table 3, Model 2, there is a significant overall indirect effect of urbanicity and MVPA while accounting for the other variables in the model, where urbanicity increases by one unit, MVPA decreases by 2.6 min (*p* = 0.05). There is also a significant direct relationship between urbanicity and MVPA, such that as urbanicity increases, MVPA decreases by 3.1 min (*p* = 0.02). In the final model for boys, perceptions of neighbourhood, social, and safety barriers did not significantly mediate the relationship between urbanicity and MVPA.

When analysed within the model, seven relationships remained significant. These relationships are seen in Table 2, Model 2. MVPA had a significant negative relation with three variables. Each year increase in age is linked with a decline in MVPA by approximately 5 min (*p* = 0.02). With each increase in urbanicity on the spectrum, MVPA decreased by 3.1 min (*p* = 0.02). In addition, with increasing perceptions of neighbourhood barriers, boys’ MVPA declined by 5.6 min (*p* = 0.04). Boys were significantly less likely to report perceptions of neighbourhood barriers as the urbanicity of their home neighbourhood increased (*p* = 0.04), and as their MHHI increased (*p* = 0.02). Reporting perception of neighbourhood barriers was significantly associated with reporting perceptions of social barriers (*p* < 0.00), as was perceiving social and safety barriers (*p* = 0.04).

## 4. Discussion

This study examined how children’s perceptions of barriers to MVPA mediates the relationship between children’s contexts and their MVPA behaviour. Previous research shows that children’s perceptions significantly differ based on their varying environments [24,32,58,59]. The present study suggests that these perceptions and urbanicity have an impact on MVPA independently, but perceptions do not mediate the relationship between urbanicity and activity levels. This study contributes to the literature by furthering the understanding of how urbanicity and barriers, impact children’s physical activity behaviours. Furthermore, to our knowledge, this was one of the first studies in the Canadian physical activity literature to utilize the spectrum approach to assessing the urbanicity of children’s home locations. With further applications, this method could prove to be a beneficial tool for assessing heterogeneous Canadian geographic contexts.

The primary hypothesis of this research was: children’s perceived neighbourhood, social, and safety barriers to physical activity would mediate the relationship of the physical environment of their neighbourhood with physical activity levels. Based on the results of the models, this hypothesis was not supported. The results indicated that barriers and the physical environment have a significant effect, and independently of each other have significant influences on MVPA, but perceptions of barriers do not mediate the relationship between the environment and physical activity in the study population.

It was further hypothesized that social barriers would have the strongest mediating effect between the physical environment and MVPA. While social barriers did not present a mediating relationship, they did independently have a significant effect on the MVPA levels of girls. Social factors and barriers influencing physical activity, especially in girls, have been well studied in the literature [59,60,61,62,63]. Qualitative research by Pawlowski et al. provides depth to this relationship, indicating girls reported having no one to play with, conflict, and peer influence of *how* social barriers influence their physical activity [61]. Given the ongoing disparities in levels of activity relative to gender, health promotion efforts must focus on alleviating social barriers in structured activity opportunities, to decrease barriers for increasing MVPA in girls [64]. Based on the evidence of Taylor et al. [32], these activities must be context-specific.

While the hypothesis that perceived neighbourhood barriers would have a mediating effect in the relationship between urbanicity and MVPA was not supported, neighbourhood barriers were significantly associated with urbanicity and MVPA for boys. The relationship between neighbourhood barriers, urbanicity, and MVPA is complex, as each are negatively associated with each other. Higher levels of MVPA are associated with lower urbanicity and perceiving fewer neighbourhood barriers. Although perceiving issues of distance, availability, and accessibility of neighbourhood resources in rural areas may not be surprising, researchers should seek to understand why boys in these areas can overcome these barriers to achieve higher levels of PA than their urban-dwelling counterparts. Furthermore, practitioners and researchers should consider the ways in which rural boys achieve more MVPA minutes than their counterparts. This may include activities not included in the traditional self-report physical activity assessments (i.e., farm chores) [65]. These findings could be a beneficial starting point for determining the disparities in activity minutes based on the home location of boys, despite the increased number of opportunities in increasingly urban settings.

Findings related to the effect of the co-variate age on MVPA were supportive of the findings in our previous work [32]. In both girls and boys, as age in years increased, minutes of MVPA decreased. This echoes recent research by Colley et al. that found the same pattern in a national sample of Canadian children [1]. While previous work determined that older children were significantly more likely than younger children to report specific barriers to physical activity, the present study did not find age significantly related to barriers when using composite scores to assess perceptions. Future work could consider examining the interplay of age in the formation of perceptions, and how this may change over time with longitudinal monitoring. In practice, it is important to develop interventions to increase MVPA in older children. As the amount of free play and physical activity during the school day decreases, health practitioners must look at strategies for engaging older children in continuation of activity habits, and provide new opportunities to continue building an appreciation for physical activity [66].

### Strengths and Limitations

This study has limitations that warrant attention. Firstly, the present study only modelled MVPA behaviour. While this is the level of behaviour recommended to achieve the maximum health benefits of activity [1], recent Canadian physical activity research has suggested considering the importance of different levels of activity and sedentary behaviour [3,8]. Future Canadian research should consider assessing the mediating effect of children’s perceptions of barriers with these multiple levels of activity to assess influences on activity achievement across the whole day. A further limitation was using dissemination area-level MHHI as the indicator for income, rather than parents’ self-report information. This could have led to a potential misrepresentation of income of the study population, and a lack of significant results. This strategy was used because a large majority of parents elected not to report their income on the parent survey, and it was not possible to impute this information. One final limitation is that there may have been measures within the model that were unaccounted for. Based on the complexity of relationships in the formation of MVPA behaviour, this will be an issue with any physical activity study. The model was based on substantive evidence in the three areas and focused on a more basic hypothesis of the mitigating relationship of perceptions, to build on the gaps of previous research.

Despite the limitations, this research laid the groundwork for future research to continue to consider the complex interaction of children’s perceptions, how they are formed within the environment, and their effect on physical activity. This study also has several strengths worth mentioning. For example, although casual inferences cannot be made for the mediating effect of perceptions on children’s physical activity in every case, it does provide a foundation for elucidating the relationship. This is of critical importance due to the conflicting results regarding the relevance of perceptions for influencing physical activity in children [67]. This was a novel and rigorous approach to assessing this relationship, using a large and diverse sample of Canadian children from two geographically distinct areas of Ontario. This study also demonstrated the possibility for using an urbanicity spectrum and the value when assessing issues related to children’s physical activity, accounting for the limitations identified in the literature related to using a dichotomous rural versus urban definition of location [50,68,69]. Finally, while a mediating relationship was not statistically significant, this study filled a gap of previous work by Taylor et al. [32], demonstrating that perceptions do have a significant influence on objectively measured MVPA. This sets the stage for future research to consider these and additional barriers in diverse environments and populations, and the implications that perceptions may have on meeting the Canadian 24-h Movement Guidelines [8].

## 5. Conclusions

The purpose of this study was to improve efforts for quantifying the experience of children’s daily activity contexts, by assessing the mediating effect of perceptions of barriers on the relationship between their environments and MVPA. These results offer insight into potential processes by which perceptions are shaped and impact MVPA and provide initial information to further the understanding of the behavioural aspects of physical activity through multiple levels of analysis. These findings suggest health promotion efforts will be most effective if they consider multipronged approaches directed toward place-specific experiences of barriers, especially targeting social barriers with girls and neighbourhood barriers with boys. While this study does not support the presence of a mediating relationship, it does support previous arguments that assessments of the objective environment are not enough to change children’s physical activity behaviour [21], and that researchers must improve efforts for quantifying the experience of children’s daily activity contexts. This work highlighted the necessity for children’s physical activity researchers in to consider new ways for assessing similarities and differences in rural and urban populations. To our knowledge, this was one of the first studies in Canadian physical activity literature to utilize the spectrum approach to assessing the relationships between urbanicity and the experience of barriers. With further applications and improvements, this method could prove as a beneficial tool for objectively assessing the heterogeneous Canadian geography, its impact on children’s experience of barriers, and their physical activity levels.

## Figures and Tables

**Table 1:** Survey questions measuring barriers, and the corresponding themed groups.

Themed Groups of Perceptions	Corresponding Survey Questions
*Neighbourhood Barriers*	Parks/playgrounds in my neighbourhood are too far from my house/takes too much time to get there
	There is not enough room at parks/playgrounds in my neighbourhood for the activities I like
	There is too much garbage/graffiti at parks/playgrounds in my neighbourhood
	There are [not] enough sidewalks on the street in my neighbourhood
	There are [no] bicycle lanes or trails in or near my neighbourhood that are easy to get to
	There are [not] a lot of trees along the streets in my neighbourhood
*Social Barriers*	There are no other kids to play with at parks/playgrounds in my neighbourhood
	I get bullied or teased when I go to parks/playgrounds in my neighbourhood
	I have nobody to go with to parks/playgrounds in my neighbourhood
	I [do not] know a lot of people in my neighbourhood
	There are too many people/it feels too crowded at parks/playgrounds in my neighbourhood
*Safety Barriers*	There is so much traffic on streets near my home that it’s difficult/unpleasant to bike or play on the street
	Most drivers go too fast while driving in our neighbourhood
	I am worried about being or walking by myself in my neighbourhood and local streets because I am worried about being taken or hurt by a stranger
	There is a lot of crime in my neighbourhood (for example, strangers, gangs, drugs)

**Table 2:** Results of the relationship between all variables within the model.

			Model 1: Girls				Model 2: Boys			
			b	se	z	*p*-Value	b	se	z	*p*-Value
Neighbourhood	on	MVPA	0.52	1.79	0.29	0.77	−5.63	2.66	−2.11	0.04 *
Social			−4.30	1.66	−2.59	0.01 *	−0.38	2.54	−0.15	0.88
Safety			0.01	0.02	0.80	0.43	0.02	0.02	0.96	0.33
Urbanicity			−0.85	0.84	−1.01	0.31	−3.10	1.31	−2.37	0.02 *
Age			−2.36	1.17	−2.01	0.04 *	−4.96	2.09	−2.38	0.02 *
MHHI			−0.53	0.50	−1.06	0.29	−0.15	0.70	−0.22	0.83
*Perceptions*										
Urbanicity	on	Neighbourhood	−0.06	0.03	−2.02	0.04 *	−0.07	0.04	−2.03	0.04 *
Age			0.01	0.04	0.34	0.74	−0.04	0.06	−0.74	0.46
MHHI			−0.03	0.02	−1.86	0.06	−0.05	0.02	−2.39	0.02 *
Urbanicity	on	Social	0.02	0.03	0.50	0.62	0.00	0.04	−0.00	0.00 **
Age			−0.02	0.05	−0.40	0.69	−0.00	0.06	−0.06	0.95
MHHI			−0.01	0.02	−0.35	0.73	−0.03	0.02	−1.27	0.21
Urbanicity	on	Safety	−0.22	3.10	−0.07	0.94	4.01	4.30	0.94	0.35
Age			6.87	4.37	1.57	0.12	0.49	6.95	0.07	0.94
MHHI			2.09	1.86	1.13	0.26	2.56	2.29	1.12	0.26
Social	with	Neighbourhood	0.29	0.04	7.59	0.00 **	0.27	0.05	6.12	0.00 **
Safety			−1.02	3.32	−0.31	0.76	−1.85	0.65	−0.40	0.69
Safety	with	Social	4.08	3.59	1.14	0.26	−10.11	4.97	−2.04	0.04 *

* Indicates significant results *p* ≤ 0.05; note: “on” signifies a one-way relationship, “with” signifies association between variables; ** Indicates significant results where *p* < 0.01. MHHI: median household income.

**Table 3:** Results of the mediating effect and direct relationships of urbanicity and perceptions on MVPA.

			Model 1: Girls				Model 2: Boys			
			b	se	z	*p*-Value	b	se	z	*p*-Value
Effects of Urban on MVPA										
*Total*			−0.95	0.84	−1.13	0.26	−2.62	1.31	−2.00	0.05 *
*Direct*	on	*MVPA*	−0.85	0.84	−1.01	0.31	−3.10	1.31	−2.37	0.02 *
*Total Indirect*			−0.10	0.18	−0.56	0.57	0.49	0.31	1.57	0.01 *
Indirect										
*Neighbourhood*	to	*MVPA*	−0.03	0.11	−0.29	0.77	0.41	0.28	1.47	0.14
*Social*	to	*MVPA*	−0.07	0.14	−0.49	0.63	0.00	0.014	0.00	1.00
*Safety*	to	*MVPA*	−0.00	0.04	−0.07	0.94	0.08	0.12	0.67	0.50

* Indicates significant results *p* ≤ 0.05; Note: “on” signifies a one-way relationship, “to” signifies mediating relationship.

## References

[1] Colley R.C., Carson V., Garriguet D., Janssen I., Roberts K.C., Tremblay M.S. (2017). Physical activity of Canadian children and youth. Health Rep..

[2] ParticipACTION (2018). The Brain + Body Equation: Canadian Kids Need Active Bodies to Build Their Best Brains.

[3] Poitras V.J., Gray C.E., Borghese M.M., Carson V., Chaput J.-P., Janssen I., Katzmarzyk P.T., Pate R.R., Connor Gorber S., Kho M.E. (2016). Systematic review of the relationships between objectively measured physical activity and health indicators in school-aged children and youth. Appl. Physiol. Nutr. Metab..

[4] Janssen I., LeBlanc A.G. (2010). Systematic review of the health benefits of physical activity and fitness in school-aged children and youth. Int. J. Behav. Nutr. Phys. Act..

[5] Strong W.B., Malina R.M., Blimkie C.J.R., Daniels S.R., Dishman R.K., Gutin B., Hergenroeder A.C., Must A., Nixon P.A., Pivarnik J.M. (2005). Evidence based physical activity for school-age youth. J. Pediatr..

[6] Warburton D.E.R., Nicol C.W., Bredin S.S.D. (2006). Health benefits of physical activity: The evidence. CMAJ Can. Med. Assoc..

[7] Jiménez-Pavón D., Kelly J., Reilly J.J. (2010). Associations between objectively measured habitual physical activity and adiposity in children and adolescents: Systematic review. Int. J. Pediatr. Obes..

[8] Tremblay M.S., Carson V., Chaput J.-P., Connor Gorber S., Dinh T., Duggan M., Faulkner G., Gray C.E., Gruber R., Janson K. (2016). Canadian 24-Hour Movement Guidelines for Children and Youth: An Integration of Physical Activity, Sedentary Behaviour, and Sleep. Appl. Physiol. Nutr. Metab..

[9] Sallis J.F., Prochaska J.J., Taylor W.C. (2000). A review of correlates of physical activity of children and adolescents. Med. Sci. Sports Exerc..

[10] Van K.D., Paw M.J., Twisk J.W., Van W.M. (2007). A brief review on correlates of physical activity and sedentariness in youth. Med. Sci. Sports Exerc..

[11] Dumith S.C., Gigante D.P., Domingues M.R., Kohl H.W. (2011). Physical activity change during adolescence: A systematic review and a pooled analysis. Int. J. Epidemiol..

[12] Wilk P., Clark A.F., Maltby A., Smith C., Tucker P., Gilliland P.A. (2017). Examining individual, interpersonal, and environmental influences on children’s physical activity levels. SSM Popul. Health.

[13] Sallis J.F., Simons-Morton B.G., Stone E.J., Corbin C.B., Epstein L.H., Faucette N., Iannotti R.J., Killen J.D., Klesges R.C., Petray C.K. (1992). Determinants of physical activity and intervention in youth. Med. Sci. Sports Excerc..

[14] Epstein L.H., Raja S., Gold S.S., Paluch R.A., Pak Y., Roemmich J.N. (2006). Reducing Sedentary Behavior.

[15] Gebremariam M.K., Altenburg T.M., Lakerveld J., Andersen L.F., Stronks K., Chinapaw M.J., Lien N. (2015). Associations between socioeconomic position and correlates of sedentary behaviour among youth: A systematic review. Obes. Rev..

[16] Ding D., Sallis J.F., Kerr J., Lee S., Rosenberg D.E. (2011). Neighborhood environment and physical activity among youth: A review. Am. J. Prev. Med..

[17] Oliveira A.F., Moreira C., Abreu S., Mota J., Santos R. (2014). Environmental determinants of physical activity in children: A systematic review. Arch. Exerc. Health Dis..

[18] Martins J., Marques A., Peralta M., Palmeira A.L., Palmeira A., Da Costa F.C. (2017). Correlates of physical activity in young people: A narrative review of reviews. Implications for physical education based on a socio-ecological approach. Retos.

[19] Tucker P., Irwin J.D., Gilliland J.A., He M. (2008). Adolescents’ Perspectives of Home, School and Neighborhood Environmental Influences on Physical Activity and Dietary Behaviors. Child Youth Environ..

[20] Tucker P., Irwin J.D., Gilliland J.A., He M., Larsen K., Hess P. (2009). Environmental influences on physical activity levels in youth. Health Place.

[21] Orstad S.L., McDonough M.H., Stapleton S., Altincekic C., Troped P.J. (2017). A Systematic Review of Agreement Between Perceived and Objective Neighborhood Environment Measures and Associations with Physical Activity Outcomes.

[22] Hume C., Salmon J., Ball K. (2004). Children’s perceptions of their home and neighborhood environments, and their association with objectively measured physical activity: A qualitative and quantitative study. Health Educ. Res..

[23] Carroll-Scott A., Gilstad-Hayden K., Rosenthal L., Peters S.M., McCaslin C., Joyce R., Ickovics J.R. (2013). Disentangling neighborhood contextual associations with child body mass index, diet, and physical activity: The role of built, socioeconomic, and social environments. Soc. Sci. Med..

[24] Davison K., Lawson C.T. (2006). Do attributes in the physical environment influence children’s physical activity? A review of the literature. Int. J. Behav. Nutr. Phys. Act..

[25] Lee H., Tamminen K.A., Clark A.M., Slater L., Spence J.C., Holt N.L. (2015). A meta-study of qualitative research examining determinants of children’s independent active free play. Int. J. Behav. Nutr. Phys. Act..

[26] Jago R., Thompson J.L., Page A.S., Brockman R., Cartwright K., Fox K.R. (2009). Licence to be active: Parental concerns and 10–11-year-old children’s ability to be independently physically active. J. Public Health (Bangk.).

[27] Sherar L.B., Gyurcsik N.C., Humbert M.L., Dyck R.F., Fowler-Kerry S., Baxter-Jones A.D. (2009). Activity and Barriers in Girls (8–16 yr) Based on Grade and Maturity Status. Med. Sci. Sports Exerc..

[28] Abraham A., Sommerhalder K., Abel T. (2010). Landscape and well-being: A scoping study on the health-promoting impact of outdoor environments. Int. J. Public Health.

[29] Beets M.W., Foley J.T. (2008). Association of Father Involvement and Neighborhood Quality with Kindergartners’ Physical Activity: A Multilevel Structural Equation Model. Am. J. Health Promot..

[30] Loebach J., Gilliland J. (2010). Child-led tours to uncover children’s perceptions and use of neighborhood environments. Child. Youth Environ..

[31] Smith F., Barker J. (2001). Commodifying the countryside: The impact of out-of-school care on rural landscapes of children’s play. Area R. Geogr. Soc..

[32] Taylor L.G., Clark A.F., Gilliland J.A. (2018). Context Matters: Examining children’s perceived barriers to physical activity across varying Canadian environments. Health Place.

[33] Wilson K., Clark A.F., Gilliland J.A. (2018). Understanding Child and Parent Perceptions of Barriers Influencing Children’s Active School Travel. BMC Public Health.

[34] Barnes J.D., Tremblay M.S. (2017). Changes in indicators of child and youth physical activity in Canada, 2005–2016. Can. J. Public Health.

[35] Nykiforuk C.I.J., Atkey K., Brown S., Caldwell W., Galloway T., Gilliland J.A., Kongats K., McGavock J., Raine K.D. (2018). Evidence synthesis—Promotion of physical activity in rural, remote and northern settings: A Canadian call to action. Health Promot. Chronic Dis. Prev. Can..

[36] Aarts M.-J., Wendel-Vos W., Van Oers H.A.M., Van De Goor I.A.M., Schuit A.J. (2010). Environmental Determinants of Outdoor Play in Children A Large-Scale Cross-Sectional Study. Am. J. Prev. Med..

[37] Walia S., Liepert B. (2012). Perceived facilitators and barriers to physical activity for rural youth: An exploratory study using photovoice. Rural Remote Health.

[38] Grow H.M., Saelens B.E., Kerr J., Durant N.H., Norman G.J., Sallis J.F. (2008). Where are youth active? Roles of proximity, active transport, and built environment. Med. Sci. Sports Exerc..

[39] Holt N.L., Neely K.C., Spence J.C., Carson V., Pynn S.R., Boyd K.A., Ingstrup M., Robinson Z. (2016). An intergenerational study of perceptions of changes in active free play among families from rural areas of Western Canada. BMC Public Health.

[40] Yousefian A., Ziller E., Swartz J., Hartley D. (2009). Active living for rural youth: Addressing physical inactivity in rural communities. J. Public Health Manag. Pract..

[41] Ryan G.J., Dzewaltowski D.A. (2002). Comparing the Relationships between Different Types of Self-Efficacy and Physical Activity in Youth. Health Educ. Behav..

[42] Ou J., Levy J., Peters J., Bongiovanni R., Garcia-Soto J., Medina R., Scammell M. (2016). A Walk in the Park: The Influence of Urban Parks and Community Violence on Physical Activity in Chelsea, MA. Int. J. Environ. Res. Public Health.

[43] Mitchell C.A., Clark A.F., Gilliland J.A. (2016). Built environment influences of children’s physical activity: Examining differences by neighbourhood size and sex. Int. J. Environ. Res. Public Health.

[44] Loebach J.E., Gilliland J.A. (2016). Free range kids? Using GPS-derived activity spaces to examine children’s neighborhood activity and mobility. Environ. Behav..

[45] Trost S.G., Pate R.R., Freedson P.S., Sallis J.F., Taylor W.C. (2000). Using objective physical activity measures with youth: How many days of monitoring are needed?. Med. Sci. Sports Exerc..

[46] Comte M., Hobin E., Majumdar S.R., Plotnikoff R.C., Ball G.D.C., McGavock J., MIPASS and Healthy Hearts Investigators Teams (2013). Patterns of weekday and weekend physical activity in youth in 2 Canadian provinces. Appl. Physiol. Nutr. Metab..

[47] Rich C., Geraci M., Griffiths L., Sera F., Dezateux C., Cortina-Borja M. (2013). Quality Control Methods in Accelerometer Data Processing: Defining Minimum Wear Time. PLoS ONE.

[48] Puyau M.R., Adolph A.L., Vohra F.A., Zakeri I., Butte N.F. (2004). Prediction of activity energy expenditure using accelerometers in children. Med. Sci. Sports Exerc..

[49] Heil D.P. (2013). Predicting Activity Energy Expenditure Using the Actical^®^ Activity Monitor. Res. Q. Exerc. Sport.

[50] Babey S.H., Tan D., Wolstein J., Diamant A.L. (2015). Neighborhood, family and individual characteristics related to adolescent park-based physical activity. Prev. Med..

[51] Brownson R.C., Chang J.J., Eyler A.A., Ainsworth B.E., Kirtland K.A., Saelens B.E., Sallis J.F. (2004). Measuring the environment for friendliness toward physical activity: A comparison of the reliability of 3 questionnaires. Am. J. Public Health.

[52] Boone H.N., Boone D.A. (2012). Analyzing likert data. J. Ext..

[53] Hoyle R.H. (1995). Structural Equation Modeling: Concepts, Issues, and Applications.

[54] Syväoja H.J., Tammelin T.H., Ahonen T., Kankaanpää A., Kantomaa M.T. (2014). The associations of objectively measured physical activity and sedentary time with cognitive functions in school-aged children. PLoS ONE.

[55] Wilk P., Clark A.F., Maltby A., Tucker P., Gilliland J.A. (2018). Exploring the effect of parental influence on children’s physical activity: The mediating role of children’s perceptions of parental support. Prev. Med..

[56] Parikka S., Mäki P., Levälahti E., Lehtinen-Jacks S., Martelin T., Laatikainen T. (2015). Associations between parental BMI, socioeconomic factors, family structure and overweight in Finnish children: A path model approach. BMC Public Health.

[57] Muthén L.K., Muthén B. (2015). Mplus: The Comprehensive Modelling Program for Applied Researchers: User’s Guide.

[58] Moore J.B., Brinkley J., Crawford T.W., Evenson K.R., Brownson R.C. (2013). Association of the built environment with physical activity and adiposity in rural and urban youth. Prev. Med..

[59] Sallis J.F., Prochaska J.J., Taylor W.C., Hill J.O., Geraci J.C. (1999). Correlates of physical activity in a national sample of girls and boys in Grades 4 through 12. Health Psychol..

[60] Dwyer J.J.M., Allison K.R., Goldenberg E.R., Fein A.J., Yoshida K.K., Boutilier M.A. (2006). Adolescent girls’ perceived barriers to participation in physical activity. Adolescence.

[61] Pawlowski C.S., Tjørnhøj-Thomsen T., Schipperijn J., Troelsen J. (2014). Barriers for recess physical activity: A gender specific qualitative focus group exploration. BMC Public Health.

[62] Spencer R.A., Rehman L., Kirk S. (2015). Understanding gender norms, nutrition, and physical activity in adolescent girls: A scoping review. Int. J. Behav. Nutr. Phys. Act..

[63] Bocarro J.N., Floyd M.F., Smith W.R., Edwards M.B., Schultz C.L., Baran P., Moore R.A., Cosco N., Suau L.J. (2015). Social and environmental factors related to boys’ and girls’ park-based physical activity. Prev. Chronic Dis..

[64] Telford R.M., Telford R.D., Olive L.S., Cochrane T., Davey R. (2016). Why Are Girls Less Physically Active than Boys? Findings from the LOOK Longitudinal Study. PLoS ONE.

[65] Davis A.M., Boles R.E., James R.L., Sullivan D.K., Donnelly J.E., Swirczynski D.L., Goetz J. (2008). Health behaviors and weight status among urban and rural children. Rural Remote Health.

[66] Gilliland J.A., Clark A.F., Tucker P., Prapavessis H., Avison W., Wilk P. (2015). The ACT-i-Pass study protocol: How does free access to recreation opportunities impact children’s physical activity levels?. BMC Public Health.

[67] McNeill L.H., Wyrwich K.W., Brownson R.C., Clark E.M., Kreuter M.W. (2006). Individual, social environmental, and physical environmental influences on physical activity among black and white Adults: A Structural Equation Analysis. Ann. Behav. Med..

[68] Jones-Smith J.C., Popkin B.M. (2010). Understanding community context and adult health changes in China: Development of an urbanicity scale. Soc. Sci. Med..

[69] Sandercock G., Angus C., Barton J. (2010). Physical activity levels of children living in different built environments. Prev. Med..

